# Measurement of Lexical Diversity in Children’s Spoken Language: Computational and Conceptual Considerations

**DOI:** 10.3389/fpsyg.2022.905789

**Published:** 2022-06-22

**Authors:** Ji Seung Yang, Carly Rosvold, Nan Bernstein Ratner

**Affiliations:** ^1^Department of Human Development and Quantitative Methodology, University of Maryland, College Park, College Park, MD, United States; ^2^Department of Hearing and Speech Sciences, Program in Neuroscience and Cognitive Science, College Park, MD, United States

**Keywords:** lexical, expressive, child language, computer-assisted, language sample analysis

## Abstract

**Background:**

Type-Token Ratio (TTR), given its relatively simple hand computation, is one of the few LSA measures calculated by clinicians in everyday practice. However, it has significant well-documented shortcomings; these include instability as a function of sample size, and absence of clear developmental profiles over early childhood. A variety of alternative measures of lexical diversity have been proposed; some, such as Number of Different Words/100 (NDW) can also be computed by hand. However, others, such as Vocabulary Diversity (VocD) and the Moving Average Type Token Ratio (MATTR) rely on complex resampling algorithms that cannot be conducted by hand. To date, no large-scale study of all four measures has evaluated how well any capture typical developmental trends over early childhood, or whether any reliably distinguish typical from atypical profiles of expressive child language ability.

**Materials and Methods:**

We conducted linear and non-linear regression analyses for TTR, NDW, VocD, and MATTR scores for samples taken from 946 corpora from typically developing preschool children (ages 2–6 years), engaged in adult-child toy play, from the Child Language Data Exchange System (CHILDES). These were contrasted with 504 samples from children known to have delayed expressive language skills (total *n* = 1,454 samples). We also conducted a separate sub-analysis which examined possible contextual effects of sampling environment on lexical diversity.

**Results:**

Only VocD showed significantly different mean scores between the typically -developing children and delayed developing children group. Using TTR would actually misdiagnose typical children and miss children with known language impairment. However, computation of VocD as a function of toy interactions was significant and emerges as a further caution in use of lexical diversity as a valid proxy index of children’s expressive vocabulary skill.

**Discussion:**

This large scale statistical comparison of computer-implemented algorithms for expressive lexical profiles in young children with traditional, hand-calculated measures showed that only VocD met criteria for evidence-based use in LSA. However, VocD was impacted by sample elicitation context, suggesting that non-linguistic factors, such as engagement with elicitation props, contaminate estimates of spoken lexical skill in young children. Implications and suggested directions are discussed.

## Introduction

Language Sample Analysis (LSA) is a time-honored tradition in the assessment of children’s expressive language skills that has been adopted as a best practice by the American Speech-Language-Hearing Association (ASHA Preferred Practice Patterns for Speech-Language Pathology)^1^, and numerous jurisdictions in the United States (Ireland and Conrad, 2016) and elsewhere, such as Europe and Asia (Oh et al., 2020; Klatte et al., 2022). However, surveys show that speech language pathologists (SLPs) derive very few measures from such analyses. Finestack et al. (2020) report that the two most frequently derived indices used by SLPs are mean length of utterance (MLU; Brown, 1973) and a vocabulary diversity measure, the Type-Token Ratio (TTR; Templin, 1957). The two presumably function together to provide a picture of a speaker’s typical utterance length and complexity, as well as the degree to which the sample shows lexical (vocabulary) richness.

### Type-Token Ratio

As noted by one of the classic texts on assessment of language production in children (Miller, 1981), “Type-token ratios are easy to compute from transcripts and provide a handy means of quantifying vocabulary” (p. 42). Calculation of the Type-Token Ratio is indeed relatively simple: the number of unique words (types) over all words in the sample (tokens). However, to be computed properly, even this requires users to collapse variations on roots (or lemmas, in current psycholinguistic parlance) as single types, a process done most easily using computer-assisted analysis such as Computerized Language Analysis (CLAN; MacWhinney, 2000) or Systematic Analysis of Language Transcripts (SALT; Leadholm and Miller, 1994). Despite its wide use, however, TTR is not without its significant detractors, almost since its introduction to the field of child language study by Templin (1957). Notably, the measure is significantly impacted by sample size, with small samples potentially showing excellent diversity simply as a function of few tokens. For instance, a poorly verbal client might say only two identical words during an interaction consisting of only 4 words, for a TTR of 0.5, considered quite high by most normative reports (Miller, 1981). Conversely, in long samples, speakers must resort to repeated use of the closed class of grammatical/function words, such as articles, prepositions and auxiliary/modal verbs, which tends to systematically decrease lexical diversity ratios over increasingly longer samples. For the record, Templin was aware of such possible influences on TTR and her own work used a standard sample size of 50 utterances in deriving reference scores for children ages 3–8 years (see Templin’s Table 60), a convention which appears to have disappeared from most contemporary discussions of the measure. However, others [e.g., Hess et al. (1986)] found no particular growth function or reliability for samples less than 350 words in length.

**FIGURE 1:**
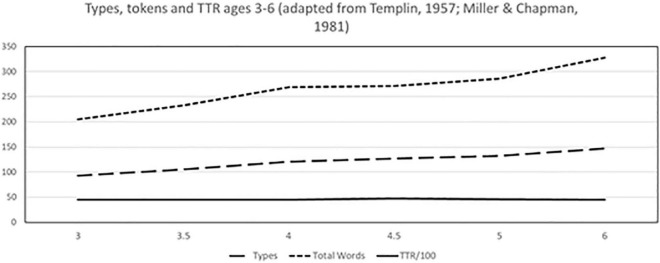
TTR as reported by Templin (1957) based on a standard 50 utterance sample.

Even when gathered over somewhat uniform sampling procedures, TTR values do not show an obvious growth trajectory; in fact, they are virtually flat, when plotted from Templin’s data (reprinted in Miller and Chapman) (see Figure 1 below). Additionally, most reference populations using TTR with preschool children are quite small in size, ranging from 480 children studied by Templin, to 69 children under age 6 in the proprietary SALT database (Leadholm and Miller, 1994). When mapped across the largest sample to date (Bernstein Ratner and MacWhinney, 2016), TTR shows an almost random trajectory over early childhood. While there have been published recommendations that TTR not be used for clinical assessment purposes (Charest and Skoczylas, 2019; Charest et al., 2020) it is inescapable that clinicians are not yet persuaded and use it frequently. Thus, we evaluate both TTR and alternatives.

### Number of Different Words

Attempts to manage the confounds inherent in TTR, when used in clinical work to contrast typical language skills with those suspected to be impaired have led to the development of a number of alternative measures to compute lexical diversity. The simplest of these essentially restores a standard sample size to the computation by selecting the number of different words in a sample of 100 words (NDW; Watkins et al., 1995). Even this threshold may be difficult to obtain from less verbal children. An alternative standardizes at 100 utterances (NDWu; Charest et al., 2020), which poses a more substantive burden on both sampling and transcription time and effort; using utterances rather than words also produces a significant confound with grammatical measures such as MLU.

Number of different words in both words and utterances has been shown to increase with age, a minimum requirement for a developmentally sensitive measure, across relatively small samples and a varying number of baseline denominators in either words (NDWw) or utterances (NDWu). However, most reference samples have been relatively small. For example, Miller et al. (1992) evaluated NDW100u in 192 typically developing children between the ages of 2:8 and just over 13 years of age, roughly 20 children at each age level. The current SALT database has reference values for NDW based on 69 preschool-aged children under 6 years of age. Klee (1992) found both a growth profile and differences between 24 pairs of typically developing (TD) children and peers with language impairment, ages 24–50 months. Watkins et al. (1995) used NDW100w to examine lexical features of samples from 75 preschool children, 25 in each of three groups (language-impaired, age-matches, and language matches). They concluded that NDW100w demonstrated both an age function as well as the ability to distinguish children with expressive vocabulary limitations for age. When the reference denominator is held constant across cohorts of children within a single study, various NDW computations have been shown to statistically differentiate certain subgroups of children in research studies, such as children with developmental language impairment (Klee, 1992; Watkins et al., 1995; Heilmann et al., 2010; Charest et al., 2020) as well as bilingual children with primary language impairment (Simon-Cereijido and Gutiérrez-Clellen, 2009). However, denominators have varied across studies that make deriving true clinical norms quite difficult. In practicality, many studies have arbitrarily defined the standard corpus length at a size that maximizes observations across groups and preserves data points [such as 41 utterances for Charest et al. (2020)]. For children older than our target population (preschool), written NDW has a moderate correlation with standardized reading vocabulary measures (Wood et al., 2019). However, attempting to create a single NDW elicitation standard for the preschool age range presents challenges: younger children (e.g., ages 2–3 years) are not able to produce reasonable narratives from prompts, and children with language delays are likely to be less talkative, making it difficult to standardize sampling sizes at numbers above 50 utterances (Yang et al., 2022) within a single diagnostic session.

### Vocabulary Diversity

A promising alternative appears to be VocD (Malvern and Richards, 2002; Malvern et al., 2004), sometimes abbreviated simply D, which uses a resampling algorithm to estimate diversity of samples differing in size. VocD is promising as an option, because it appears to show growth trajectories over childhood in two studies (Malvern et al., 2004; Bernstein Ratner and MacWhinney, 2016) and correlated well with standardized vocabulary test scores in one investigation (Silverman and Bernstein Ratner, 2002). VocD has been shown to distinguish between children with language impairment in both English (Owen and Leonard, 2002) and Chinese in some studies (Klee et al., 2004; Wong et al., 2010; Wu et al., 2019). However, even when used as part of a composite score with MLU, the sensitivity of VocD was less than optimal (Wong et al., 2010).

Additionally, VocD is not without its own limitations. First, although it has differentiated some clinical samples from typically developing children (Tager-Flusberg et al., 1990; Silverman and Bernstein Ratner, 2002) it does show small variations as a result of sample size (Owen and Leonard, 2002). Second, it must be computed using a computer algorithm, something that most practicing clinicians appear to avoid, to date (Finestack and Satterlund, 2018). Finally, as with the other measures we discuss, VocD does not have a deep literature suggesting its clinical validity as a measure that can help professionals to identify children who do not meet developmental benchmarks.

### Moving Average TTR

The most recently developed lexical diversity measure is the moving average TTR (*MATTR*; McCarthy and Jarvis, 2010). Like VocD, MATTR must be computed using a computer algorithm. This measure has been used to evaluate the very short samples typically generated by adults with aphasia (Harris Wright et al., 2003; Fergadiotis et al., 2013) and one large-scale study of children ages 4–9 years (Charest et al., 2020) that used a standard elicitation procedure (Edmonton Narrative Norms Instrument) as well as a study of bilingual children with language impairment (Kapantzoglou et al., 2017). In that study, MATTR (using a window of 50 words) was the best distinguishing measure of lexical diversity, accounting for 98% of the variance, while VocD accounted for 73% and TTR much less at 32% of the variance.

Frustratingly, this and other studies have used a wide variety of sampling window sizes, typically set at the length of the shortest sample produced by individual study participants; for example, a window as low as 17 tokens for patients with aphasia (Fergadiotis et al., 2013) and as high as 100 words for typically developing and developmentally language delayed children (Charest et al., 2020). As window sizes vary, so do resulting MATTR scores (Kapantzoglou et al., 2019). This is certainly acceptable for research studies contrasting two groups of participants using a uniform window size for resampling, but makes determination of an optimal sample size impossible at this time. For trial purposes, we will examine two potential window sizes for MATTR.

Thus, although efforts have been made to validate measures of children’s expressive vocabulary since the 1950’s, few comprehensive efforts have been made to answer some questions basic to the use of any of these diversity measures in child assessment. These include:

Do any of the three most commonly referenced measures of children’s expressive vocabulary diversity show a consistent pattern of growth over the preschool years (ages 2–6 years) when computed across a much larger number of children than previously surveyed? A profile of growth with age would seem to be a minimum requirement for a clinically valid measure of language use (Channell et al., 2018). Additionally, we sought to employ a standard, predetermined window for NDWw (NDW100w) that was reasonable for hand computation by the typical working clinician.

Critical to their common use in clinical language assessment, do any of the diversity measures reliably distinguish children thought to be typically developing from children known to have expressive language impairment?

Do the three measures appear to index the same behaviors (e.g., are they interchangeable for clinical and research purposes)? If so, NDW would show an advantage in cases where a faster, non-computer-assisted measure is desirable.

We also ask two additional questions:

Do any of the three measures appear to be influenced by non-lexical factors? As noted by others (Charest et al., 2020) valid measures of lexical diversity should not be confounded by other expressive language measures, such as mean length of utterance.

These questions are addressed in Study 1.

A last question, which appears not to have been examined in any detail to date, is whether lexical diversity measures are an epiphenomenon of the contexts in which language samples are collected. Most prior work has simply asked whether children’s lexical profiles are determined by genre [e.g., conversational vs. narrative (Westerveld et al., 2004; Channell et al., 2018)]. Popescu (2009) described TTR as “not a measure of richness, but a measure of information flow, topic deployment” (p. 233). If so, we should be able to find associations between lexical diversity measures and the availability of props or toys even within a genre, such as conversation. We address this question in Study 2.

Some research has suggested that lexical diversity measured by VocD does differ by task even within a general genre, such as story telling compared to story retell (Kapantzoglou et al., 2017). This, in turn, would suggest a need for standardizing language sampling contexts even within conversational interaction, a concept we have recently discussed in relation to another measure of child language growth, the Index of Productive Syntax (IPSyn; Yang et al., 2022).

## Study 1: Large N Characteristics of Typically Used Measures of Lexical Diversity in Children

### Materials and Methods

Data that were analyzed in this study are derived from the TalkBank CHILDES archive, North America (NA) at RRID:SCR_003241.

#### Participants

Participants were taken from CHILDES corpora in English/North America (NA) and the Clinical MOR English NA subdirectory. These are the same samples used by Yang et al. (2022), with one exception: no children with Hearing Loss were included in the current analysis due to extreme heterogeneity in lexical profiles on all measures we were studying. The number of samples per study and the descriptive statistics for gender and age for these three groups of children are presented in Table 1.

**TABLE 1:** Number of language samples per study and the children sample characteristics across three groups.

Group	CHILDES	Studies	*N* of corpora	100utt criterion satisfied		50utt criterion satisfied	
		Bates	101	0		7	
		Bliss	7	7		7	
		Morisset	196	0		53	
		NewmanRatner/24	124	4		59	
	Eng-NA	Tardif	25	0		0	
		Valian	43	36		37	
Toy/Cross/Typical		VanHouten (freeplay)	45	3	*N* = 338;	22	*N* = 639;
		VanKleeck	40	31	Mean Age = 40.4;	36	Mean Age = 38.0;
		Warren	20	13	52% male	17	47% male
		EllisWeismer/TD	296	126		271	
		Feldman/ParentChild/TD	57	22		28	
	Clinical-MOR	Hooshyar/TD/play	29	0		0	
		Nicholas/TD	103	68		71	
		Rondal/TD	41	28		31	
		EllisWeismer/LT	280	68		221	
		Rescorla	70	38	*N* = 126;	55	*N* = 354;
Toy/Long/Atypical	Clinical-MOR	EisenbergGuo/LT	17	16	Mean Age = 48.6;	49	Mean Age = 45.8;
		Hargrove	82	0	68% male	4	70% male
		UCSD-SLI	55	4		25	
An error in the conversion from LaTeX to XML has occurred here. 2*Down syndrome	An error in the conversion from LaTeX to XML has occurred here. 2*Clinical-MOR	Hooshyar-DS-play	31	1	*N* = 16;	4	*N* = 27;
		Rondal-DS	41	15	Mean Age = 53.6; 56.3% male	23	Mean Age = 49.2; 55.6% male

This resulted in a sample size of 1,454 transcripts of speech between children ages 2 and 6 years, all of which were cross-sectional in primary design (although a few studies had made yearly re-analyses, these are contrasted with longitudinal, dense sampling of few children, such as Brown’s Adam, Eve and Sarah). Critically, all were unstructured sessions involving the target child with a single adult in toy play. This can be contrasted with corpora in which children were engaged with other children, during snack or mealtime, or prompted using a standard elicitation task, such as picture description or story retell.

##### Sample Selection

We think it worthwhile to note that some children’s samples did not permit use of many lexical diversity options because they failed to meet a baseline number of utterances; only TTR does not require a threshold, which may be why clinicians continue to use it. We, however, set a minimum number of 10 words in a child sample to be included in the analyses reported for TTR. VocD asks for 50 token samples; NDW by definition asks for 100. Because no guidelines exist to set MATTR window size for young children, we arbitrarily chose two values under 50 utterances (10, 25) to see whether such small windows [comparable to those used in measuring the speech of people with aphasia (PWA)] to standard prompts would function well in less controlled elicitation settings.

Because of differing requirements for sample size upon which to compute averages, numbers of eligible samples differed widely across ages and cohorts (typical, delayed/impaired). For each outcome analysis, we used all available cases not to lose the representation of the population as well the statistical power. We have noted specific numbers of observations as appropriate in reporting results.

#### Analysis

To examine the pattern of growth over the preschool years (ages 2–6 years) for each measure, various regression models that include linear, linear-linear segmented regression, and curve linear regression models were fitted. These parametric model results were also visually contrasted to non-parametric regression model results to find the best representation of the data. Once the best model was decided, we contrasted the fitted lines or curves between typically developing children and delayed children to examine the possibility of utilizing each measure diagnostically to distinguish language delay or disorder from typical developmental profiles. Finally, bivariate correlations for each group and across ages were calculated and examined among lexicon diversity measures as well as their relation to other non-lexical indices such as MLU and IPSyn scaled scores.

### Results

#### Growth Trajectory

**FIGURE 2:**
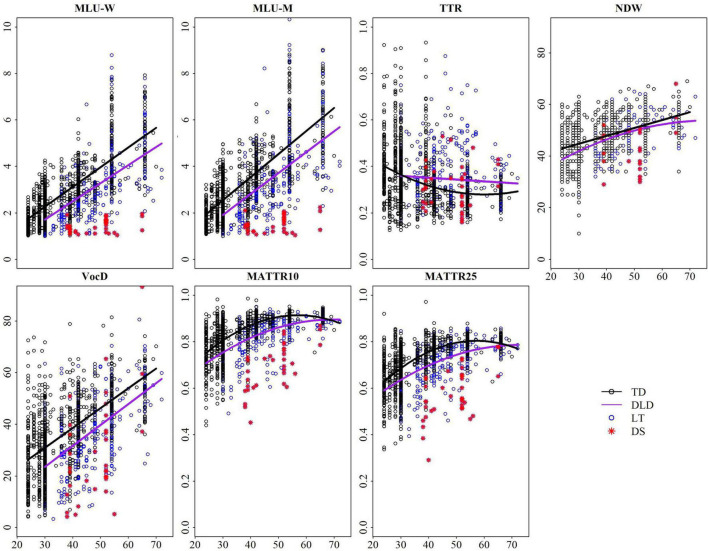
Final fitted trajectory of each measure across age for (Typically developing; TD and Developmentally language delayed; DLD) groups. DLD subtypes are broken down further to Late Talking (LT) children and those with Down Syndrome (DS).

As shown in Figure 2, which depicts the final regression model for each measure, TTR shows no discernable growth profile in either typical or language-impaired preschool children. Conversely, both NDW and VocD do show growth trajectories, at least for children considered typically developing. In the case of NDW, trajectories for typical and impaired children do not diverge significantly until children approach 72 months of age. For VocD, the profile for children closely mirrors that of MLU, showing a steady growth function in typical children, late talking children and children with Down Syndrome. Finally, MATTR values (at both window sizes) show a curvilinear function, plateauing and then declining after about 4 years of age.

#### Ability to Distinguish Between Typical and Atypical Development

For this cohort of children, only VocD provides reliable information about overall status linguistically at all time points from 2 to 6 years of age. TTR actually would suggest that typically developing children function less well than their language-impaired peers. NDW provides poor sensitivity through the age range, as profiles are minimally distinct across groups of children. Consistent with the aphasia literature, which has turned to MATTR for analysis of short, sparse language samples, MATTR shows some differentiation between groups before about 4 years of age, but values merge for children over this age.

#### Are Vocabulary Measures Interchangeable?

**FIGURE 3:**
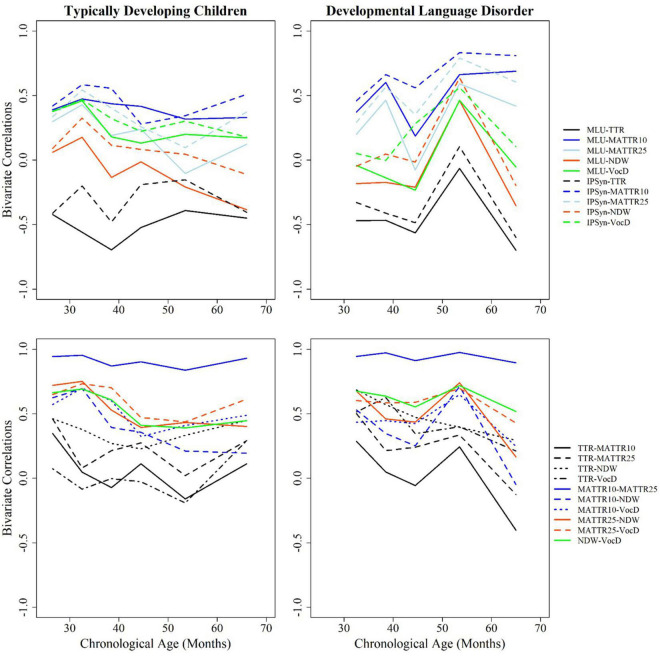
Bivariate correlations among measures across ages by group.

Inter-correlations for the entire cohort once more strongly counter-indicate use of TTR for clinical purposes. It is only weakly correlated with the other two vocabulary measures, and has a higher, albeit inverse correlation with known measures of grammatical development. This suggests that, as has been previously suggested, mature use of free grammatical morphemes (articles, verb auxiliaries/modals, prepositions, conjunctions, etc.) can depress vocabulary diversity scores. In contrast, NDW and VocD are highly intercorrelated (0.7) with each other, but not with TTR (see Table 2). We have also created visual displays of change in correlation values over the preschool age range in Figure 3.

**TABLE 2:** Bivariate correlations among measures.

	MLU_W	MLU_M	IPsynT	TTR	MATTR10	MATTR25	NDW	VocD
**TD**								
MLU_W	946	**1.00**	**0.85**	−**0.50**	**0.59**	**0.51**	**0.29**	**0.57**
MLU_M	946	946	**0.86**	−**0.50**	**0.60**	**0.53**	**0.30**	**0.57**
IPsynT	635	635	635	−**0.30**	**0.73**	**0.68**	**0.41**	**0.61**
TTR	946	946	635	946	−0.06	0.04	**0.24**	−**0.15**
MATTR10	946	946	635	946	946	**0.96**	**0.65**	**0.70**
MATTR25	934	934	635	934	934	934	**0.71**	**0.75**
NDW	757	757	634	757	757	757	757	**0.70**
VocD	880	880	635	880	880	880	757	880
**DLD**								
MLU_W	390	**0.99**	**0.86**	−**0.27**	**0.64**	**0.55**	**0.33**	**0.43**
MLU_M	390	390	**0.85**	−**0.27**	**0.63**	**0.55**	**0.32**	**0.40**
IPsynT	390	390	390	−**0.15**	**0.78**	**0.72**	**0.48**	**0.56**
TTR	390	390	390	390	**0.13**	**0.26**	**0.43**	**0.34**
MATTR10	390	390	390	390	390	**0.97**	**0.63**	**0.63**
MATTR25	390	390	390	390	390	390	**0.70**	**0.70**
NDW	390	390	390	390	390	390	390	**0.73**
VocD	390	390	390	390	390	390	390	390

*The bold upper diagonal numbers are significant correlations p < 0.05 and the lower diagonal numbers are corresponding sample sizes. Statistically insignificant correlations are in gray.*

#### Are Vocabulary Measures Confounded by Grammatical Growth?

We have presented correlations in two sets: the top figures relate lexical measures to grammatical measures, while the bottom set relate lexical measures to each other. TTR and MATTR are the most highly correlated with syntactic measures (MLU and IPSyn), but in opposite directions. TTR is negatively related to syntactic development, while MATTR appears to reflect it. In general, although results vary somewhat along the preschool age span, NDW and VocD show negligible correlation with syntactic measures, a desirable trait for a measure that purports to reflect only vocabulary range. In the bottom frames, we see that both values of MATTR are highly inter-correlated, suggesting that window size does not play a major role in computing the measure. TTR shows the lowest correlations with other vocabulary measures, possibly because it does not show a regular function in our analyses. Finally, the two most highly inter-correlated measures other than MATTR10/MATTR25 are NDW and VocD, suggesting that both tap into similar constructs.

### Discussion, Study 1

Of the major traditional and emerging measures of lexical diversity in young children’s expressive language, only VocD meets the challenges of an evident growth profile over the age range, and ability to distinguish between typically developing children and those with known expressive language impairment. NDW shows a growth profile, but little ability to distinguish between groups. Of all measures examined, TTR is by far the weakest, and should not be used for either research or clinical purposes. It does not demonstrate any relationship to chronological age in children from the ages 2 to 6 years, and in fact characterizes children with expressive language disorder as more lexically diverse in their spoken language than typically developing children. This finding is not novel, and the major concern is that texts, researchers and clinicians do not appear to heed repeated demonstration of its inherent weakness as an expressive language measure. Finally, if the goal in using a lexical diversity measure is to measure lexical richness itself, rather than a by-product of grammatical growth, then VocD and NDW are preferred, as they do not show significant inter-correlation with traditional measures of expressive syntax (MLU in either words or morphemes); in contrast, together with their other weaknesses, TTR and MATTR are highly confounded by other aspects of child language development.

## Study 2: Are Vocabulary Measures Used in LSA Confounded by Elicitation Context?

### Materials and Methods

#### Participants

This study utilized data sourced from the FluencyBank English UMD-CMU corpus of the CHILDES Database (MacWhinney, 2000). These children had been recruited as controls for a larger study that examined children’s fluency and language skills longitudinally for 3 years. Additional controls from late-talking and Spanish-English bilingual cohorts were also used in the current study, however, their data are not yet publicly available. Participants included in the analysis were 32 typically developing children (22 males) between the ages of 29 and 50 months (*M* = 36.2, SD = 5.9).

#### Language Samples and Testing

Video recordings of spontaneous language samples elicited between the children and their parents while engaged in joint play with a standard set of props (e.g., building blocks, doctor’s kit, play food, dolls) were transcribed according to CHAT conventions (MacWhinney, 2000). Videos were subsequently reviewed to identify the number of meaningful elicitation props used by the parent-child dyad in each session. A battery of receptive and expressive standardized assessments was administered by the original researchers. Of these scores, the participants’ *Peabody Picture Vocabulary Test* (*PPVT-4*; Dunn, 2018) Raw Scores were included as a proxy for general vocabulary development.

### Analysis

#### Spontaneous Language Measures

For this analysis, we only included TTR because it is most highly used by clinicians (e.g., per Finestack and Satterlund, 2018) and VocD, because it emerged as the strongest measure of lexical diversity in Study 1. All measures were derived in the same manner using CLAN KidEval utilities as in Study 1.

#### Analysis of Lexical Diversity

To assess factors that influenced TTR and VocD, multiple regressions with number of props, MLU- m, and *PPVT Raw Score (PPVT RS)* as predictors was run. Raw scores were used for *PPVT* because many children were under the normative range for the instrument. We measured and included as covariates factors that we expected to have some relationship with lexical diversity, MLU in morphemes (MLU-m), which was included as a proxy for general grammatical development and *PPVT-4* Raw Score, which was included as a proxy for general vocabulary development.

### Results

**FIGURE 4:**
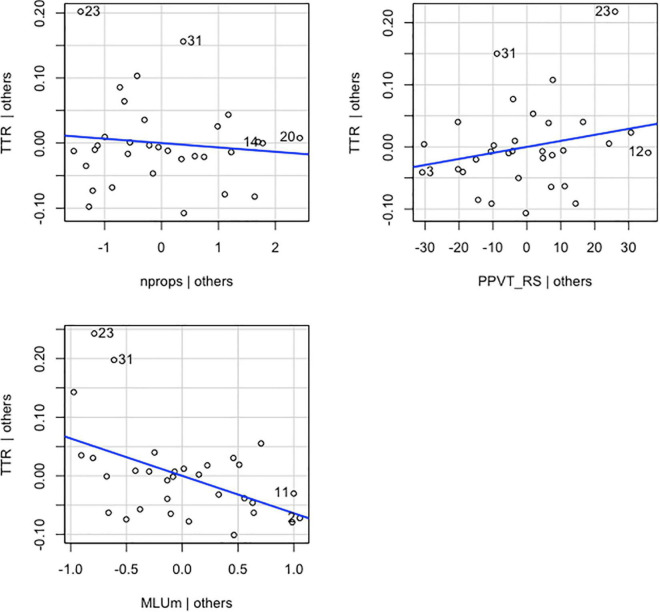
Added-variable plots of individual explanatory variables (nprops, MLUm and *PPVT*_RS) versus the dependent variable (TTR) and their linear fit (blue line).

For TTR scores regressed on MLU-m, number of props, and *PPVT* RS, the overall multiple regression was statistically significant [*R*^2^ = 0.247, *F*(3,28) = 3.069, *p* < 0.05]. The three variables accounted for 25% of the variance in TTR. However, the only independent variable to have a statistically significant effect on TTR, above and beyond the other two predictors, was MLU-m. The unstandardized regression coefficient (β) for MLU-m was −0.064 [*t*(28) = −2.949, *p* = 0.006], meaning that for each additional MLU-m, children’s TTR decreased by 0.064, controlling for *PPVT* RS and number of props. β associated with the number of props was −0.007 [*t*(28) = 0.012, *p* = 0.570]. This finding suggests that for each additional toy prop that was a focus of interaction, TTR will decrease by 0.007, controlling for MLU-m and *PPVT* RS. These results suggest that although the overall model is significant, only MLU-m is indeed an important influence on TTR above and beyond the other two predictors. Graphical representations of the partial regression model depicting the relationship between the main coefficient tested and the dependent variable along with its covariates are shown in Figure 4.

**FIGURE 5:**
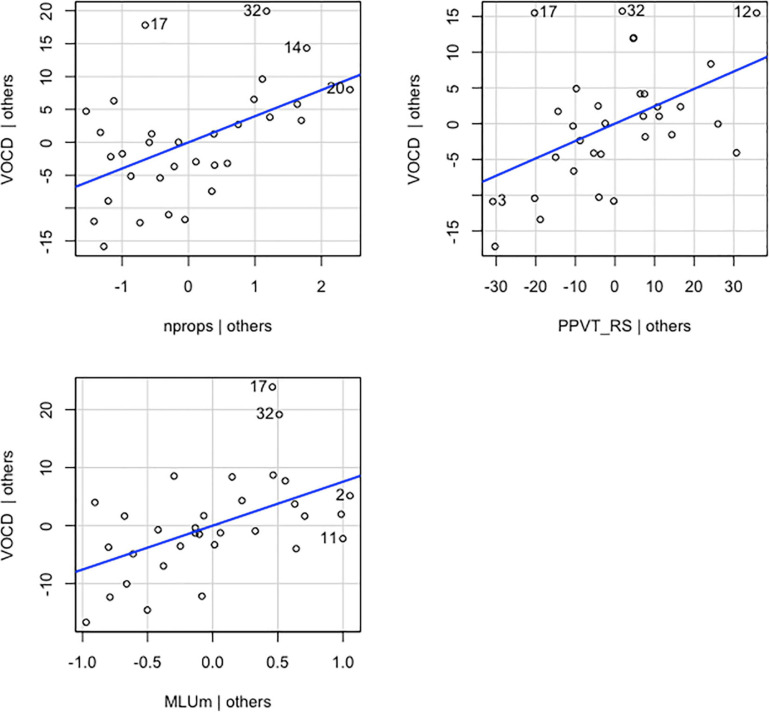
Added-variable plots of individual explanatory variables (nprops, MLUm and *PPVT*_RS) versus the dependent variable (VOCD) and their linear fit (blue line).

Vocabulary diversity scores were regressed on the same predictors as the previous model (MLU-m, number of props, and *PPVT* RS). The overall multiple regression was statistically significant [*R*^2^ = 0.618, *F*(3,28) = 15.07, *p* < 0.001]. The three variables accounted for 62% of the variance in VocD. All three independent variables had a statistically significant effect on VocD, above and beyond the other two predictors. The unstandardized regression coefficient (β) for MLU-m was 7.570 [*t*(28) = 3.151, *p* = 0.004], meaning that for each additional MLU-m, children’s VocD increased by 7.570, controlling for *PPVT* RS and number of props. β associated with the number of props was 3.979 [*t*(28) = 3.071, *p* = 0.005]. This finding suggests that for each additional prop that a child interacts with, their VocD will increase by 3.979, controlling for MLU-m and *PPVT* RS. These results suggest that the overall model and each of its predictors have an important influence on VocD scores above and beyond the other two predictors. Graphical representations of the partial regression model depicting the relationship between the main coefficient tested and the dependent variable along with its covariates are shown in Figure 5.

A Pearson correlation was used to address the relationship between TTR (*M* = 5.00, SD = 2.98) and VocD (*M* = 5.00, SD = 2.58) in this smaller sample of language samples. As with the larger analysis reported above, the two measures were weakly and negatively correlated [*r*(30) = −0.18, *p* = 0.314 (two-tailed)].

### Discussion Study 2

In this study, we assessed whether different lexical diversity measures are vulnerable to the influence of preference or allocation of focus toward props on the part of the parent or child. We found that the number of elicitation props meaningfully played with was a significant predictor of VocD. This means that language samples of parent-child free play contexts including more switches between toys had higher lexical diversity measures. These results provide direct evidence that non-linguistic factors, such as the number of elicitation props used in a language sample, can capture major lexical information. More specifically, the results demonstrate how assessments of a child’s language from unguided interactions such as parent-child play can be manipulated by factors of child or parent interest in props.

These findings illuminate two important implications for language sampling. First, although there is little prior literature to suggest an impact of lab props or child/adult toy preference in obtaining lexical diversity scores, our preliminary results provide compelling evidence that non-linguistic factors capture viable lexical information and are not an insubstantial determinate of lexical diversity. As in our larger analysis reported first, different measures of lexical diversity produce differing profiles. This suggests a need for large-scale evaluation of lexical diversity measures that are more robust, developmentally sensitive, or immune to contextual influences.

## Discussion

### Comparing Vocabulary Diversity Measures

Our work here confirms a blunt assessment made by Vermeer (2000), who minced no words when she called TTR “the worst measure of lexical richness” (p. 69). Charest et al. (2020) pronounced TTR “disfavored for clinical use” (p. 1868) and not worthy of research investigation. Thus, it is dispiriting to note its frequent use in clinical practice (Finestack and Satterlund, 2018), in texts that educate clinicians in training, and even recent research articles examining measurement of expressive child language skills (Gonzalez Villasanti et al., 2020; Manning et al., 2020; Kubota et al., 2021; Arjmandi et al., 2022). In this study, across more than 1500 conversational language samples from children ages 2 to 6 years of age, TTR was worse than uninformative: if used to make clinical decisions, it would have identified numerous children with known impairment as typically functioning, while identifying otherwise typical children as deficient in expressive lexical diversity during conversation. It also showed either an inverse relationship to age (typical children) or was unaffected by age (language delayed children).

The findings for NDW100w were mixed. As in many other studies, it showed a clear trajectory by age for both typical and language delayed children. However, its ability to differentiate between typical and language-delayed children was virtually nil at most age points, and showed a large degree of overlapping scatter.

Of the two computer algorithms, VocD, which has shown promising results when applied to large cohorts of typically developing children (Malvern et al., 2004; Bernstein Ratner and MacWhinney, 2016) was indeed sensitive to chronological age, and was capable of distinguishing between typical and atypically developing children. Results were more mixed for MATTR at two window settings (10 and 25 words): while trends were distinct for group and age prior to 50 months, both growth and discriminative function fell above that age range.

It should also be noted by visual inspection of our regression plots that individual variation was extensive for all five measures, perhaps in part due to our concerns about confounding variables that we discuss in Study 2. Low sensitivity to disorder might be counterbalanced by the ability of a measure to guide intervention. We and others have discussed this concept previously (Overton et al., 2021; Yang et al., 2022) in relationship to some grammatical analyses of spoken language samples by indices such as Developmental Sentence Scoring (Lee, 1974) or the Index of Productive Syntax (Altenberg et al., 2018). In each of these cases, low diagnostic sensitivity can easily be balanced by the relatively rich information it can provide to the intervention process (e.g., Finestack et al., 2020; Pezold et al., 2020).

However, as an informative index upon which to build intervention, it is not clear how any of these lexical diversity measures improves upon information provided by any standardized test of vocabulary understanding or production, since both sets of measures are meant to infer size of the child’s available lexicon using a limited set of items as a proxy indicator of all words known by the child. Our general conclusion from our analyses here is that LSA measures of vocabulary diversity, unless narrowly constrained by elicitation genre and even physical number of elicitation prompts, contribute little to our diagnostic or therapeutic recommendation processes beyond those provided by vocabulary tests or parental checklists (e.g., MCDI), which at least have the advantage of standardized administration (even if likely to be confounded by child experience, dialect variation and socioeconomic status).

For solutions that recommend a standard sample size in words or utterances, Vermeer (2000) also noted that in her work, as in others, total sample size in words grows with child age. Thus, truncating samples to a standard number of tokens or even utterances will cause the problem that we investigate in Study 2, that of context on resulting diversity measures. As sentences grow in length, the number of topics that can be discussed within a set sample window will decline, decreasing likely variability in the number of different words used.

What might therapists do in order to gauge children’s lexical skills? If standardized vocabulary tests are meant to serve as proxies for lexical knowledge, then they may be most appropriate to use for identification purposes. Even parental report of vocabulary size for children too young to test directly on standardized tests is a well-established and normed process for identification of children who are delayed at language onset (Makransky et al., 2016; Mayor and Mani, 2019; DeMayo et al., 2021). However, if a goal of language sample analysis is to identify not only children’s relative level of expressive language skill, but also to guide intervention, clinicians may want to focus not only the variety of lexical forms used, but also its complexity (as potentially measured by computer algorithms that estimate relative frequency of use in the language, based on appropriate word frequency estimates for the preschool population). Or they may wish to focus on whether word use is appropriate/in error, as suggested by Charest and Skoczylas (2019); these authors identified language delay in a small sample of 14 children via analysis of lexical errors, despite no differences in NDW. Or they may wish to consider that all children benefit from vocabulary enrichment during intervention, regardless of initial testing results (Bleses et al., 2016).

In painting this somewhat dismal picture of traditional measures of lexical diversity used in preschool conversational LSA, we wish to note that we are not rejecting its use with other populations and in other contexts. We are currently replicating this set of analyses with large corpora of child narratives, and we think it highly likely that one or more measures may perform well when children respond to a standardized set of prompts in either spoken or written form. However, it is precisely because very young children, especially those who are already considered at risk, may not perform well in such contexts that LSA has assumed greater importance in the diagnostic process. It is in such uncontrolled contexts and at the narrow age points we identify (ages 2–5 years) that we do not see impeccable psychometric properties for the lexical metrics we discuss in this article. The best of these measures (VocD) requires clinicians to use computer-assisted algorithms. It was still contaminated by testing context.

In sum, given the set of findings presented here, until we identify more LSA measures that appear to perform well diagnostically, we suspect that both fair and informative language sample analysis for preschool aged children may need to rethink common recommendations to adjust the elicitation process for each child, merely to increase the child’s likelihood of conversing with the parent or adult interlocutor. This call (see also Yang et al., 2022) to re-envision and standardize LSA prompts for both lexical and syntactic analysis would run directly counter to some currently advised by LSA specialists, who advise that “the play context can be adjusted to meet individual preferences, individual interests, gender, culture and experience” in the service of the largest possible sample (Miller et al., 2015, p. 13–14). While laudable in principle and client-centered focus, we do not currently appear to have lexical diversity measures for preschool children’s LSA that satisfy basic requirements of such measures for either diagnostic or therapy planning purposes. We believe that such measures may be more reliable under conditions typically used with older children, such as elicited narrative or story retell, in which prompts remain stable from sample to sample. For example, recent large N research suggests that NDW in kindergarteners’ elicited narrative can be stable and capable of predicting later language skills two grades later (Murphy et al., 2022). It is to such types of diagnostic LSA samples that we address in our ongoing research.

## Data Availability Statement

The original contributions presented in this study are included in the article/supplementary material, further inquiries can be directed to the corresponding author.

## Author Contributions

All authors listed have made a substantial, direct, and intellectual contribution to the work, and approved it for publication.

## Conflict of Interest

NB was a consultant to federal grants that support the development of the CLAN utilities used in data analysis reported here. The remaining authors declare that the research was conducted in the absence of any commercial or financial relationships that could be construed as a potential conflict of interest.

## Publisher’s Note

All claims expressed in this article are solely those of the authors and do not necessarily represent those of their affiliated organizations, or those of the publisher, the editors and the reviewers. Any product that may be evaluated in this article, or claim that may be made by its manufacturer, is not guaranteed or endorsed by the publisher.

## Footnotes

1
https://www.asha.org/policy/pp2004-00191/#sec1.3.13

## References

[B1] Altenberg E. P. Roberts J. A. Scarborough H. S. (2018). Young children’s structure production: a revision of the index of productive syntax. *Lang. Speech Hear. Serv. Sch.* 49 995–1008. 10.1044/2018_LSHSS-17-0092 29978201 PMC6430505

[B2] Arjmandi M. K. Houston D. Dilley L. C. (2022). Variability in quantity and quality of early linguistic experience in children with cochlear implants: evidence from analysis of natural auditory environments. *Ear Hear.* 43 685–698. 10.1097/AUD.0000000000001136 34611118 PMC8881322

[B3] Bernstein Ratner N. B. MacWhinney B. (2016). Your laptop to the rescue: using the child language data exchange system archive and CLAN utilities to improve child language sample analysis. *Semin. Speech Lang.* 37 074–084. 10.1055/s-0036-1580742 27111268 PMC8856510

[B4] Bleses D. Makransky G. Dale P. S. Højen A. Ari B. A. (2016). Early productive vocabulary predicts academic achievement 10 years later. *Appl. Psycholinguist.* 37 1461–1476.

[B5] Brown R. (1973). *A first language: The early stages.* Cambridge, MA: Harvard University.

[B6] Channell M. M. Loveall S. J. Conners F. A. Harvey D. J. Abbeduto L. (2018). Narrative language sampling in typical development: implications for clinical trials. *Am. J. Speech Lang. Pathol.* 27 123–135. 10.1044/2017_AJSLP-17-0046 29222570 PMC6105083

[B7] Charest M. Skoczylas M. J. (2019). Lexical diversity versus lexical error in the language transcripts of children with developmental language disorder: different conclusions about lexical ability. *Am. J. Speech Lang. Pathol.* 28 1275–1282. 10.1044/2019_AJSLP-18-0143 31335160

[B8] Charest M. Skoczylas M. J. Schneider P. (2020). Properties of lexical diversity in the narratives of children with typical language development and developmental language disorder. *Am. J. Speech Lang. Pathol.* 29 1866–1882. 10.1044/2020_AJSLP-19-00176 32692626

[B9] DeMayo B. Kellier D. Braginsky M. Bergmann C. Hendriks C. Rowland C. F. (2021). *Web-CDI: A System for Online Administration of the MacArthur-Bates Communicative Development Inventories. Language Development Research.* Available online at: https://web.archive.org/web/20201105043625id_/http://www.e-csd.org/upload/csd-25-2-411.pdf$^{*}$ad (accessed May 28, 2022).

[B10] Dunn D. M. (2018). *Peabody Picture Vocabulary Test—V.* Boston, MA: NCS Pearson.

[B11] Fergadiotis G. Wright H. H. West T. M. (2013). Measuring lexical diversity in narrative discourse of people with aphasia. *Am. J. Speech Lang. Pathol.* 22 S397–S408. 10.1044/1058-0360(2013/12-0083 23695912 PMC3813439

[B12] Finestack L. H. Satterlund K. E. (2018). Current practice of child grammar intervention: a survey of speech-language pathologists. *Am. J. Speech Lang. Pathol.* 27 1329–1351. 10.1044/2018_AJSLP-17-0168 30458473

[B13] Finestack L. H. Rohwer B. Hilliard L. Abbeduto L. (2020). Using computerized language analysis to evaluate grammatical skills. *Lang. Speech Hear. Serv. Sch.* 51 184–204. 10.1044/2019_LSHSS-19-00032 32255745 PMC7225022

[B14] Gonzalez Villasanti H. Justice L. M. Chaparro-Moreno L. J. Lin T.-J. Purtell K. (2020). Automatized analysis of children’s exposure to child-directed speech in reschool settings: validation and application. *PLoS One* 15:e0242511. 10.1371/journal.pone.0242511 33237919 PMC7688182

[B15] Harris Wright H. Silverman S. Newhoff M. (2003). Measures of lexical diversity in aphasia. *Aphasiology* 17:443.

[B16] Heilmann J. J. Miller J. F. Nockerts A. (2010). Using language sample databases. *Lang. Speech Hear. Serv. Sch.* 41 84–95. 10.1044/0161-1461(2009/08-0075) 20051580

[B17] Hess C. W. Sefton K. M. Landry R. G. (1986). Sample size and type-token ratios for oral language of preschool children. *J. Speech Lang. Hear. Res.* 29, 129–134. 10.1044/jshr.2901.129 3702374

[B18] Ireland M. Conrad B. J. (2016). Evaluation and eligibility for speech-language services in schools. *Perspect. ASHA Spec. Interest Groups* 1 78–90.

[B19] Kapantzoglou M. Fergadiotis G. Auza Buenavides A. (2019). Psychometric evaluation of lexical diversity indices in Spanish narrative samples from children with and without developmental language disorder. *J. Speech Lang. Hear. Res.* 62 70–83. 10.1044/2018_JSLHR-L-18-0110 30950757

[B20] Kapantzoglou M. Fergadiotis G. Restrepo M. A. (2017). Language sample analysis and elicitation technique effects in bilingual children with and without language impairment. *J. Speech Lang. Hear. Res.* 60 2852–2864. 10.1044/2017_JSLHR-L-16-0335 28915297

[B21] Klatte I. S. van Heugten V. Zwitserlood R. Gerrits E. (2022). Language sample analysis in clinical practice: speech-language Pathologists’ barriers, facilitators, and needs. *Lang. Speech Hear. Serv. Sch.* 53 1–16. 10.1044/2021_LSHSS-21-00026 34694898

[B22] Klee T. (1992). Developmental and diagnostic characteristics of quantitative measures of children’s language production. *Top. Lang. Disord.* 12 28–41.

[B23] Klee T. Stokes S. F. Wong A. M. Y. Fletcher P. Gavin W. J. (2004). Utterance length and lexical diversity in Cantonese-speaking children with and without specific language impairment. *J. Speech Lang. Hear. Res.* 47 1396–1410. 10.1044/1092-4388(2004/104) 15842018

[B24] Kubota M. Chondrogianni V. Clark A. S. Rothman J. (2021). Linguistic consequences of toing and froing: factors that modulate narrative development in bilingual returnee children. *Int. J. Biling. Educ. Biling.* 1–19. *v, 10.1080/13670050.2021.1910621

[B25] Leadholm B. J. Miller J. F. (1994). *Language Sample Analysis: The Wisconsin Guide. Bulletin 92424.* Milwaukee, WI: Wisconsin State Department of Public Instruction.

[B26] Lee L. L. (1974). *Developmental Sentence Analysis: A Grammatical Assessment Procedure for Speech and Language Clinicians.* Evanston, IL: Northwestern University Press.

[B27] MacWhinney B. (2000). *Tools for Analyzing Talk Part 2: The CLAN Program.* Pittsburgh, PA: Carnegie Mellon University.

[B28] Makransky G. Dale P. S. Havmose P. Bleses D. (2016). An item response theory–based, computerized adaptive testing version of the macarthur–bates communicative development inventory: words and sentences (CDI: WS). *J. Speech Lang. Hear. Res.* 59 281–289. 10.1044/2015_JSLHR-L-15-0202 27050253

[B29] Malvern D. Richards B. (2002). Investigating accommodation in language proficiency interviews using a new measure of lexical diversity. *Lang. Test.* 19 85–104.

[B30] Malvern D. Richards B. Chipere N. Durán P. (2004). *Lexical Diversity and Language Development: Quantification and Assessment.* London: Palgrave Macmillan. 253. 10.1057/9780230511804

[B31] Manning B. L. Harpole A. Harriott E. M. Postolowicz K. Norton E. S. (2020). Taking language samples home: feasibility, reliability, and validity of child language samples conducted remotely with video chat versus in-person. *J. Speech Lang. Hear. Res.* 63 3982–3990. 10.1044/2020_JSLHR-20-00202 33186507 PMC8608210

[B32] Mayor J. Mani N. (2019). A short version of the MacArthur–Bates communicative development inventories with high validity. *Behav. Res. Methods* 51 2248–2255. 10.3758/s13428-018-1146-0 30306410

[B33] McCarthy P. M. Jarvis S. (2010). MTLD, vocd-D, and HD-D: a validation study of sophisticated approaches to lexical diversity assessment. *Behav. Res. Methods* 42 381–392. 10.3758/BRM.42.2.381 20479170

[B34] Miller J. (1981). *Assessing Language Production in Children: Experimental Procedures.* Boston, MA: Allyn and Bacon.

[B35] Miller J. F. Andriacchi K. Nockerts A. (2015). *Assessing Language Production Using SALT Software: A Clinician’s Guide to Language Sample Analysis*, 2nd edn. Middleton, WI: SALT Software.

[B36] Miller J. F. Freiberg C. Rolland M. B. Reeves M. A. (1992). Implementing computerized language sample analysis in the public school. *Top. Lang. Disord.* 12, 69–82. 10.1097/00011363-199202000-00008

[B37] Murphy K. A. Springle A. P. Sultani M. J. McIlraith A. Language and Reading Research Consortium (Larrc)[Corporate Author]. (2022). Predicting language performance from narrative language samples. *J. Speech Lang. Hear. Res.* 65 775–784. 10.1044/2021_JSLHR-21-00262 34990557

[B38] Oh S. J. Yoon J. H. Lee Y. (2020). A qualitative study on experiences and needs of language sample analysis by speech–language pathologists: focused on children with language disorders. *Commun. Sci. Disord.* 25 169–189.

[B39] Overton C. Baron T. Pearson B. Z. Ratner N. B. (2021). Using free computer-assisted language sample analysis to evaluate and set treatment goals for children who speak African American English. *Lang. Speech Hear. Serv. Sch.* 52 31–50. 10.1044/2020_LSHSS-19-00107 33464988 PMC8711707

[B40] Owen A. J. Leonard L. B. (2002). Lexical diversity in the spontaneous speech of children with specific language impairment: application of D. *J. Speech Lang. Hear. Res.* 45 927–937. 10.1044/1092-4388(2002/075) 12381050

[B41] Pezold M. J. Imgrund C. M. Storkel H. L. (2020). Using computer programs for language sample analysis. *Lang. Speech Hear. Serv. Sch.* 51 103–114. 10.1044/2019_LSHSS-18-0148 31697609

[B42] Popescu I.-I. (2009). *Word Frequency Studies.* Berlin: De Gruyter Mouton.

[B43] Silverman S. Bernstein Ratner N. (2002). Measuring lexical diversity in children who stutter: application of vocd. *J. Fluency Disord.* 27 289–303. 10.1016/s0094-730x(02)00162-6 12506447

[B44] Simon-Cereijido G. Gutiérrez-Clellen V. F. (2009). A cross-linguistic and bilingual evaluation of the interdependence between lexical and grammatical domains. *Appl. Psycholinguist.* 30 315–337. 10.1017/S0142716409090134 19444336 PMC2681320

[B45] Tager-Flusberg H. Calkins S. Nolin T. Baumberger T. Anderson M. Chadwick-Dias A. (1990). A longitudinal study of language acquisition in autistic and Down syndrome children. *J. Autism Dev. Disord.* 20 1–21. 10.1007/BF02206853 2139024

[B46] Templin M. C. (1957). *Certain Language Skills in Children: Their Development and Interrelationships*, Vol. 10. Minneapolis, MN: University of Minnesota Press.

[B47] Vermeer A. (2000). Coming to grips with lexical richness in spontaneous speech data. *Lang. Test.* 17 65–83.

[B48] Watkins R. V. Kelly D. J. Harbers H. M. Hollis W. (1995). Measuring children’s lexical diversity: differentiating typical and impaired language learners. *J. Speech Lang. Hear. Res.* 38 1349–1355. 10.1044/jshr.3806.1349 8747826

[B49] Westerveld M. F. Gillon G. T. Miller J. F. (2004). Spoken language samples of New Zealand children in conversation and narration. *Adv. Speech Lang. Pathol.* 6 195–208. 10.3109/17549507.2016.1159332 27142252

[B50] Wong A. M.-Y. Klee T. Stokes S. F. Fletcher P. Leonard L. B. (2010). Differentiating Cantonese-speaking preschool children with and without SLI using MLU and lexical diversity (D). *J. Speech Lang. Hear. Res.* 53 794–799. 10.1044/1092-4388(2009/08-0195) 20530389 PMC7251334

[B51] Wood C. L. Bustamante K. N. Schatschneider C. Hart S. (2019). Relationship between children’s lexical diversity in written narratives and performance on a standardized reading vocabulary measure. *Assess. Eff. Interv.* 44 173–183. 10.1177/1534508417749872 34045929 PMC8153412

[B52] Wu S.-Y. Huang R.-J. Tsai I.-F. (2019). The applicability of D, MTLD, and MATTR in Mandarin–speaking children. *J. Commun. Disord.* 77 71–79. 10.1016/j.jcomdis.2018.10.002 30686328

[B53] Yang J. S. MacWhinney B. Ratner N. B. (2022). The Index of productive syntax: psychometric properties and suggested modifications. *Am. J. Speech Lang. Pathol.* 31 239–256. 10.1044/2021_AJSLP-21-00084 34748390 PMC9135028

